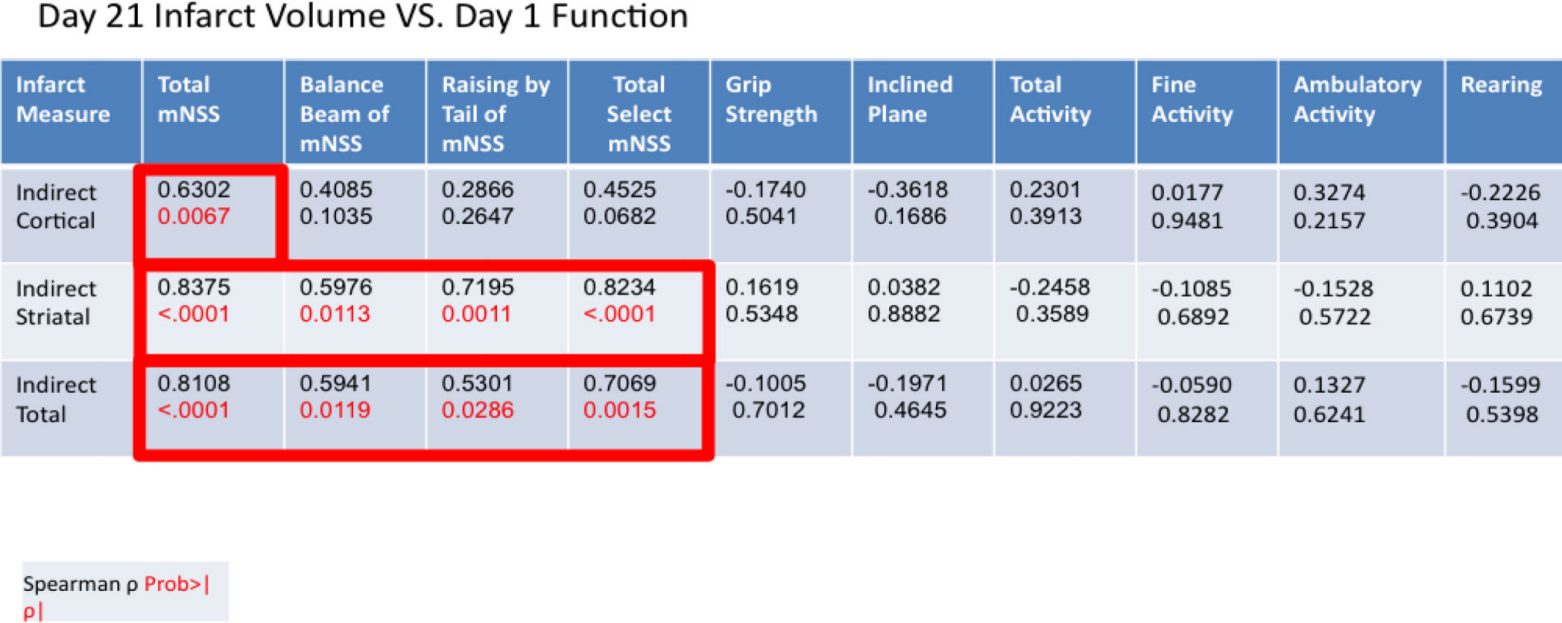# The role for infarct volume as a surrogate measure of functional outcome following ischemic stroke

**DOI:** 10.15761/JSIN.1000136

**Published:** 2016-10-11

**Authors:** Ryan C Turner, Kenneth DiPasquale, Aric F Logsdon, Zhenjun Tan, Zachary J Naser, Jason D Huber, Charles L Rosen, Brandon P Lucke-Wold

**Affiliations:** 1 Department of Neurosurgery, School of Medicine, West Virginia University, Morgantown, West Virginia 26506, USA; 2 Center for Neuroscience, School of Medicine, West Virginia University, Morgantown, West Virginia 26506, USA; 3 Department of Basic Pharmaceutical Sciences, School of Pharmacy, West Virginia University, Morgantown, West Virginia 26506, USA

**Keywords:** ischemic stroke, behavior, functional outcome

## Abstract

The failed translation of proposed therapeutic agents for ischemic stroke from preclinical to clinical studies has led to increased scrutiny of preclinical studies, namely the model and outcome measures utilized. Preclinical studies routinely use infarct volume as an experimental endpoint or measure in studies employing young-adult, healthy male animals despite the fact that clinically, ischemic stroke is a disease of the elderly and improvements in functional outcome from pre- to post-intervention remains the most widely utilized assessment. The validity of infarct volume as a surrogate measure for functional outcome remains unclear in clinical studies as well as preclinical studies, particularly those utilizing a more clinically relevant aged thromboembolic model. In this work, we will address the relationship between acute and chronic functional outcome and infarct volume using a variety of functional assessments ranging from more simplistic, subjective measurements such as the modified Neurologic Severity Score (mNSS), to more complex, objective measurements such as grip strength and inclined plane.

## Introduction

Ischemic stroke remains a leading cause of morbidity and mortality worldwide and is associated with significant direct and indirect costs. Present treatment of ischemic stroke is limited to thrombolysis, achieved via either mechanical or pharmacological means, both of which are limited to a small cohort of patients based on time of presentation after stroke onset and an extensive list of contraindications. Consequently, there remains a large unmet need for either alternative treatment options or a means for increasing eligibility for thrombolysis. The development of a successful neuroprotectant, since discovery of the ischemic penumbra by Astrup and colleagues [1–3], has been the focus of extensive research in ischemic stroke and produced over 100 candidates progressing to clinical trials based on the work of over 1000 preclinical studies [4]. Unfortunately, none of these proposed neuroprotective agents have translated from bench-to-bedside successfully.

Reasons for the failed translation remain speculative; however advisory groups composed of leaders in the field have identified numerous potential reasons for the lack of successful translation [5–8]. These include the animal models and outcome measures utilized. The vast majority of preclinical studies have employed healthy, young-adult male animals, despite the fact that stroke is largely a disease of the elderly [5,9]. The aged brain not only responds to an insult or injury differently but also exhibits less restorative capacity in comparison to the healthy and young brain [9–14]. Additionally, preclinical studies emphasize histological outcome measures, namely infarct volume quantification, whereas the focus of clinical studies is on functional assessments and restoration of physical and cognitive skills [5,15]. How infarct volume relates to functional outcome is a subject of controversy with clinical studies demonstrating a moderate correlation (Spearman’s rho of 0.43 – 0.59 depending on the study) between final infarct volume and function assessed using common scales of neural injury [16,17]. Preclinical studies have demonstrated a multitude of findings depending on the model of ischemia, functional assays performed, method of correlation, and time points assessed post- ischemia [18–26]. These findings are difficult to interpret due to the aforementioned variables as well as the use of generally healthy, young-adult animals in the vast majority of studies, a population that fails to replicate the clinical population afflicted with ischemic stroke.

In this work we investigate for the first time ever in an aged thromboembolic model of ischemia and tPA-mediated reperfusion the relationship between infarct volume and functional outcome. Specifically, we address the following questions: 1) do acute decrements in functional ability following ischemia correlate with the location or size of the infarct; 2) do chronic deficits in function equate to larger infarct volumes; 3) do acute functional deficits correlate with larger infarcts chronically; and 4) does the time point of assessment indicate the inclusion or exclusion of certain functional assessments?

We seek to address these questions in what we believe represents a more clinically relevant preclinical model of ischemic stroke. The use of functional assessments is emphasized in clinical studies and therefore needs to be considered in preclinical work. By incorporating the use of a functional assessment array in a more relevant model of ischemia, we address for the first time using this model the relevance of histological measures as a surrogate measure of outcome. This work illustrates the importance of functional ability as an outcome measure in preclinical ischemic stroke studies on the basis of more closely replicating clinical experience.

## Methods

All chemicals used in this study were of molecular biology grade and purchased from Sigma Aldrich (St. Louis, MO), unless stated otherwise. Human recombinant tPA was gifted by Genentech (San Francisco, CA). Female Sprague-Dawley rats were acquired from Hilltop Animal Laboratory (Scottsdale, PA). All animals used in this work were obtained between 18–21 months of age from an aging colony established at the supplier. Animals were housed under 12 hour light/12 hour dark conditions with food and water available ad libitum. Animals were acclimated for a minimum of 7 days prior to all experimental procedures. The West Virginia University Animal Care and Use Committee approved all experiments.

### Surgery: Middle cerebral artery occlusion (MCAO)

Rats were anesthetized using inhaled isoflurane (4% induction, 2% maintenance) from Halocarbon (River Edge, NJ) and underwent thromboembolic MCAO as previously described [10]. Reperfusion was achieved through administration of human recombinant tPA (5 mg/kg i.v., femoral vein; 30% bolus and 70% infused over 30 minutes using a syringe drive). Ischemia was defined as a perfusion drop across the MCA territory of >80% as determined by laser Doppler measurements. Successful reperfusion was considered upon restoration of blood flow to >80% of baseline values within 30 minutes of tPA administration. The time of tPA administration was varied from 2–6 hours to produce ischemia of a multitude of severities and consequently, infarcts of different sizes. Rats were then divided into acute (n=47) and chronic assessment (n=17) groups with the acute group being sacrificed at 24 hours post-MCAO and the chronic group at 21 days post-MCAO.

### Behavioral testing

All functional testing was performed at baseline to obtain a baseline value for normalization on an animal-by-animal basis based on previous experience of the authors and the potential for weight and size-related effects. There was no more than 1 week between baseline testing and induction of MCAO. Following MCAO, testing occurred at 24 hours, 72 hours, 7 days, 14 days, and 21 days in the chronic assessment paradigm and only at 24 hours in the acute paradigm, immediately prior to sacrifice.

#### Modified Neurological Severity Score (mNSS)

The mNSS, described previously by Watanabe and colleagues, is a widely used method for assessing the severity of functional deficits after neurological injury in rodents [15]. The test includes measures of sensorimotor performance and basic reflex integrity. The balance beam used was a synthetic composite material and possessed a square profile with rounded corners and measured 4 × 4 cm.

#### Grip Strength

The Grip Strength Meter, a commercially available product, was acquired from Columbus Instruments. This apparatus is composed of a load cell and interchangeable grip attachment. This work utilized the T-bar attachment. A total of 5 measures were taken for forelimb grip strength for each rat and averaged to produce a composite score at each time point. The rats were grasped by the tail and nape of the neck, forepaws placed onto the bar, and pulled in a straight line away from the load cell by gripping the animal at the base of the tail.

### Activity monitoring

Activity was recorded using an automated monitoring system from San Diego Instruments (San Diego, CA). Rats were given 1 hour to acclimate to the testing room prior to initiation of testing. Each chamber for testing consisted of a Plexiglass housing surrounded by a photobeam array (16 × 16) to detect movements in the x-y plane. A separate 8 photobeam array detected rearing activity. Activity was recorded over a period of 30 minutes and included measures of ambulatory, fine, rearing, and total activity in increments of 5 minutes.

### Inclined plane

An inclined plane was constructed to model that described by Rivlin and Tator [15]. The device consisted of an 18 × 18 cm platform with a textured, rubberized coating. The platform was affixed to a base and hinged on one side, allowing rotation from the horizontal to 85°. Rats were placed on the apparatus such that the main axis of the body was perpendicular to the inclined plane. The maximum incline angle at which the rat could successfully maintain its position for 5 seconds was recorded as the plane was elevated slowly. As the plane is elevated, the rat can make corrective adjustments to maintain a normal posture using both fore- and hind limbs. Rats were subjected to 5 trials on the inclined plane per assessment period with these measurements being averaged to again produce a composite score.

### Histology

At the time of sacrifice, animals in the acute group were saline perfused prior to brain extraction and subsequent staining with 2% triphenyl tetrazolium chloride (TTC) as previously described [9]. The chronic group was perfused with 4% paraformaldehyde prior to brain extraction and immersion in fixative for a minimum of 24 hours. Brains were then processed using a Tissue-Tek VIP 5 automatic processor and paraffin embedded using a Tissue-Tek TEC 5 embedding console system, both of which were acquired from Sakura Finetek (Torrance, CA). Paraffin-embedded tissues were sectioned at 6 m using a Leica RM2235 microtome (Richmond, VA). Alternating sections were stained for infarct volume assessment using standard protocols for hematoxylin & eosin (H&E) and cresyl echt violet. Briefly, sections were deparaffinized using xylene and rehydrated through a series of alcohol immersions progressing from 100% ethanol to 70% ethanol. For H&E, sections were immersed in hematoxylin solution for 2.25 minutes, rinsed in running tap water, and dipped in a 0.25% acid alcohol solution to differentiate. Slides were again rinsed in running tap water and blued in Scott’s Tap Water Substitute for one minute prior to immersion in 95% ethanol for 30 seconds and subsequent counterstaining with Eosin Y for 30 seconds. For cresyl echt violet, sections were stained in in cresyl echt violet solution (pH 2.5) for 4 minutes prior to dehydration and clearance with xylene. These techniques allow for assessment of infarct volume despite the presence of significant gliosis at chronic time points.

### Infarct volume quantification

For quantification of TTC stained sections, images were acquired using a standard flatbed scanner and quantified using Adobe Photoshop. For quantification of H&E and cresyl echt violet stained sections, images were acquired using an Olympus AX-70 microscope and quantified using Image J. A blinded observer performed all quantification.

### Statistical analysis

All data was analyzed using JMP 10 from the SAS Institute (Cary, NC). Correlations between infarct volume and functional performance were assessed by the Spearman rank-sum coefficient. Differences are considered to be statistically significant at the P<0.05 level.

## Results

Acute impairments in functional performance on the mNSS following MCAO in aged rodents correlate with striatal infarct volume.

**Figure 1:**
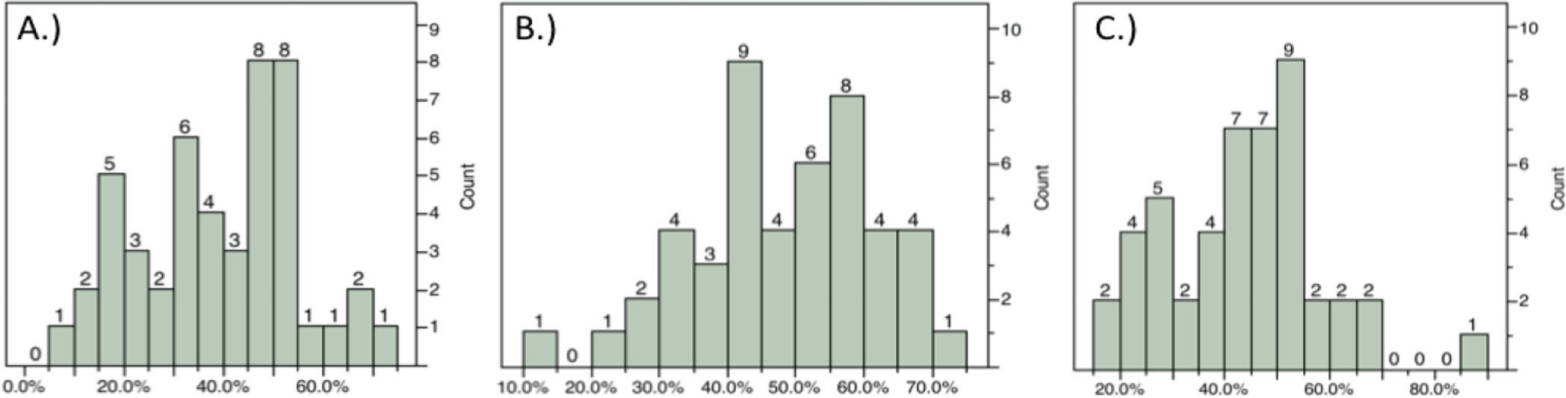
Distribution of infarct volumes by region at 24h using TTC staining. Distribution of infarct volumes produced within the cortex A), striatum B), and total C) in this study obtained through variation of the length of ischemia.

At 24 hours after MCAO, rats underwent functional assessment using the mNSS, grip-strength meter, inclined plane, and recording of spontaneous activity immediately prior to sacrifice and infarct volume determination using TTC staining. The distribution of TTC staining was determined from each rat for the ipsilateral cortex, striatum, and total hemisphere (Figure 1). A moderate correlation was observed based on Spearman rank-order correlation between total mNSS score and indirect striatal infarct volume (ñ = 0.4455, P = 0.0031) as well as the balance beam and rising by the tail subtests of the mNSS (ñ = 0.4147, P = 0.0063; ñ = 0.3596, P = 0.0193; respectively). Activity monitoring revealed a significant decline in the number of rearings recorded post-stroke with a moderate correlation between rearing and striatal infarct volume observed (ρ = 0.4523, P = 0.0121).

Interestingly, both total mNSS and subtests of the mNSS universally failed to correlate with cortical and total infarct volumes at 24 hours post-MCAO (Table 1).

While mNSS failed to correlate with striatal and cortical infarct volume in the acute period, both inclined plane and grip strength indicated a strong trend towards correlation with cortical (ρ = 0.4379, P = 0.0535; ρ = −0.4081, P = 0.0663) and total (ρ = 0.4230, P = 0.0631; ρ = −0.4205, P = 0.0577) infarct volumes but not striatal infarcts (Table 1).

The relationship between the various functional assessments and the edema index was also determined as at 24 hours post-MCAO, significant edema is present and may influence outcome as evident by the numerous proposed and tested therapeutic agents targeting various elements of ischemic stroke pathophysiology associated with edema development. Significant correlations were observed between total mNSS (ρ = 0.3145, P = 0.0452) and the inclined plane (ρ = 0.5445, P = 0.0159).

Total mNSS scores correlate with cortical, striatal, and total infarct volume when assessed at 21 days post-MCAO.

**Figure 2:**
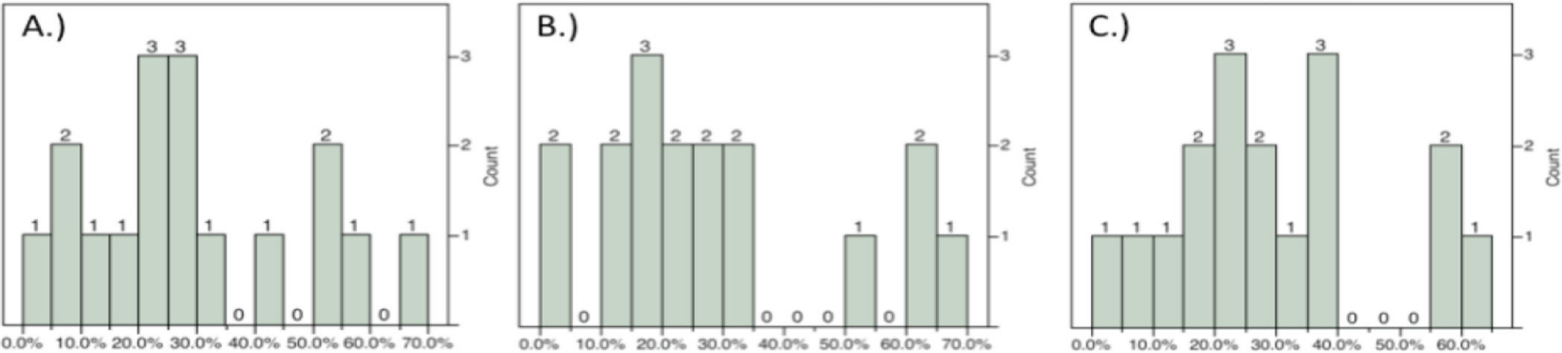
Distribution of infarct volumes by region at 21d using H&E staining. Distribution produced within the cortex A), striatum B), and total C) in this study obtained through variation of the length of ischemia.

**Figure 3:**
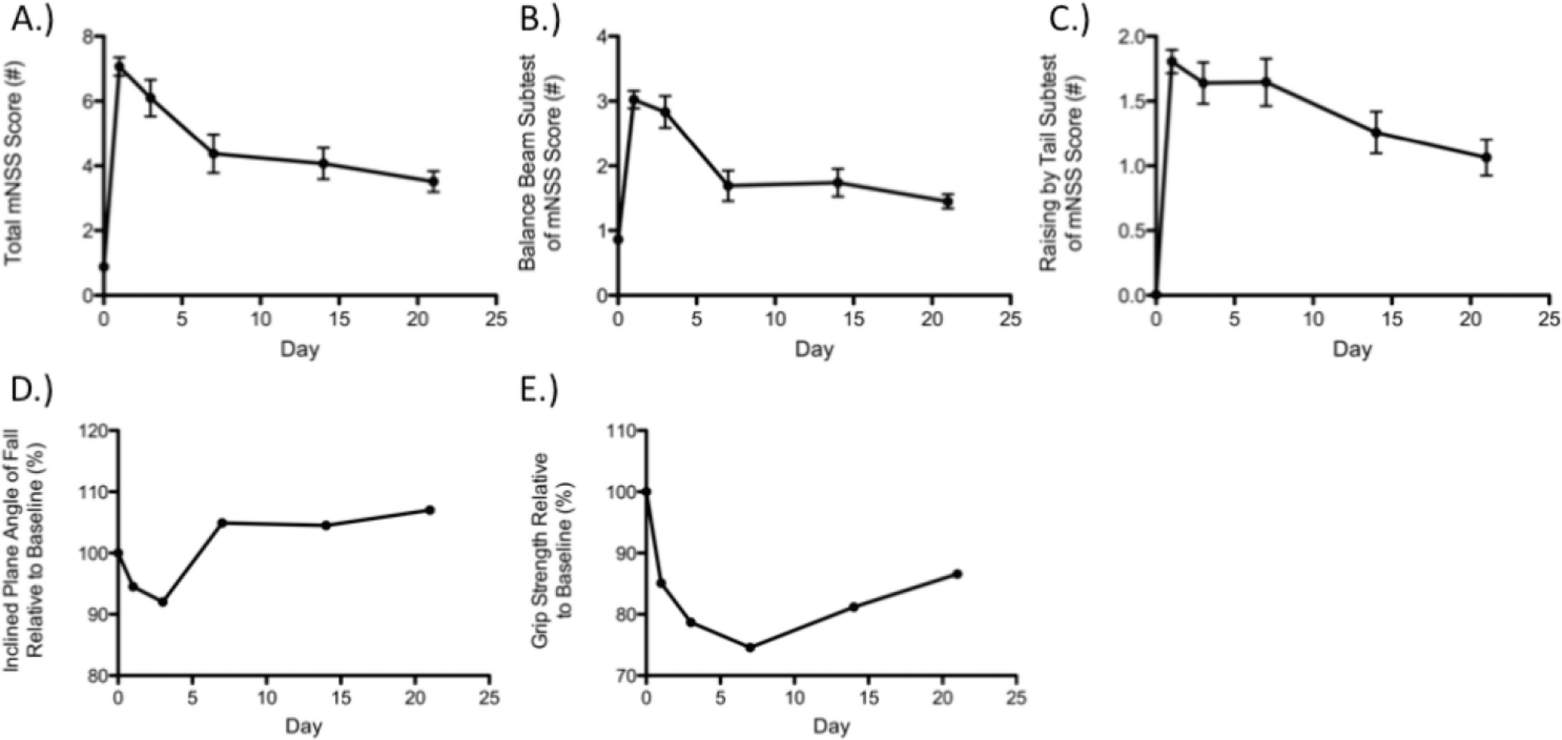
Demonstration of functional recovery occurring over three weeks after MCAO with regards to total mNSS A), beam balance subtest of the mNSS B), raising by the tail subtest of the mNSS C), inclined plane D), and grip-strength E).

Functional assessments were performed on Day 21 post-MCAO immediately prior to sacrifice and infarct volume determination using hematoxylin and eosin (H&E) staining (Figure 2). Moderate to strong correlations were observed between total mNSS and cortical (ρ = 0.5590, P = 0.0196), striatal (ρ = 0.6187, P = 0.0081), and total (ρ = 0.6988, P = 0.0018) infarct volumes. Subtests such as the balance beam and raising by the tail also correlated with various elements of infarct volume when both function and histology were assessed at 21 days post-MCAO (Table 2). Specifically, performance on the balance beam subtest of the mNSS correlated with cortical and total infarct volumes (ρ = 0.7457, P = 0.0060; ρ = 0.7457, P = 0.0006; respectively) but not striatal. Raising of the tail, another subtest of the mNSS, was related to striatal (ρ = 0.6200, P = 0.0079) and total (ρ = 0.5404, P = 0.0251) infarct volumes but not cortical (Table 2). No other assessments detected deficits or impairments at day 21 consistent with infarct volume (Table 2). The reasons for this are unclear but likely include the gradual return of function with increased time post-MCAO to a level frequently near, or approximating, the baseline (Figure 3). The lack of significant correlation at these time points is therefore not entirely surprising but indicates a need for more sensitive functional measures following MCAO that display persistent deficits, a particular problem in the likely less relevant but far more widely used young-adult models of ischemic stroke. Similarly, the plasticity of the rodent brain that allows for functional recovery (Figure 3) may necessitate an array of functional assessments dependent on time post-infarct of interest in order to capture both initial deficits and return of function prior to saturation (generally baseline).

Acute deficits determined using the mNSS may predict cortical, striatal, and total infarct volumes at 21 days post-MCAO in aged rodents.

Moderate to strong correlations were observed between total mNSS and cortical (ρ = 0.6302, P = 0.0067), striatal (ρ = 0.8375, P < 0.0001), and total (ρ = 0.8108, P < 0.001) infarct volumes in animals with functional performance assessed at 24 hours post-MCAO and infarct volumes determined histologically at day 21 post-ischemia. Subtests such as the balance beam and raising by the tail also correlated with striatal (ρ = 0.5976, P = 0.0113; ρ = 0.7195, P = 0.0011) and total (ρ = 0.5941, P =0.0119; ρ = 0.5301, P = 0.0286) infarct volume but not cortical infarction when function was assessed at 24 hours post-MCAO and histology was assessed at 21 days post- MCAO (Table 3). No other assessments correlated with any of the infarct measures (Table 3).

## Discussion

Failure of successful bench-to-bedside translation of proposed therapeutics for ischemic stroke, namely neuroprotectants, has resulted in an extensive array of recommendations for preclinical studies going forward. Some of the most notable and agreed upon guidelines include the incorporation of functional and histological endpoints, prolonged survival times post-MCAO, and perhaps most importantly, the consideration of more clinically relevant models that address aging and/or comorbidities frequently afflicting patients with ischemic stroke. This work shows for the first time in a thromboembolic model of ischemic stroke using aged animals, the relationship between infarct volume and functional impairment at both acute and chronic time points. Moderate to strong correlations were observed between neurological scoring systems such as the mNSS and infarct volume at numerous time points. Perhaps most notable is the strength and persistence of this correlation between total mNSS score and striatal infarct volumes at both day 1 and day 21. This finding is in contrast to comparisons between total mNSS and cortical infarct volumes in which no correlation was observed at 24 hours post-MCAO between function and volume but a moderate to strong correlation was found at 21 days post-MCAO and when using day 1 function as a predictor of day 21 infarct volume.

Why cortical infarct volumes do not correlate with function acutely remains unclear but potential reasons include the impact of edema acutely following MCAO and disruption of the functional circuitry as a consequence of striatal damage regardless of cortical integrity. Edema occurs rapidly following ischemia and subsequent blood-brain barrier disruption, likely reaching a maximum within the first five days post- ischemia and has been shown to be associated with various neurological deficits identified in otherwise healthy young-adult, male rats [17,27]. These findings are consistent with work presented here in which edema correlated with the total mNSS score and inclined plane. Perhaps more importantly, the striatum serves as a relay for numerous connections related to motor and sensorimotor function in the rat. The striatum, composed of the caudate nucleus, putamen, and ventral striatum (a combination of the nucleus accumbens and surrounding areas), receives afferent connections from the cortex, substantia nigra, and thalamic nuclei (centromedian and parafascicular). Efferent connections originating in the striatum pass to the thalamus and substantia nigra, in addition to numerous intra-striatal connections (caudate to the putamen, putamen to the globus pallidus, etc). Consequently, any lesion within the striatum can alter function of other brain regions and result in anatomical changes outside of the ischemic territory such as thalamic shrinkage. A large striatal stroke may disrupt transmission to and from the cortex, regardless of cortical health, thereby diminishing the likelihood of correlation between cortical infarct size and functional deficits. Therefore, impairments in function are likely regardless of cortical health, diminishing the likelihood of strong volume-function correlations with regards to cortical and total volumes acutely [28]. This is particularly true with regards to the aged rodent model utilized herein as cortical volumes are typically larger in this model due to reduce collateralization with age and therefore, increased tissue susceptibility to ischemia [29]. There is less discrepancy in collateral flow with regards to the striatum between young and aged rodents, resulting in more uniformity when considering striatal infarct volumes. Delayed assessments post-stroke, such as those conducted within this work, generally display an improvement or recovery in function, a finding consistent with significant plasticity of the rodent brain.

While the inclusion of functional assessments has been identified as a recommended experimental endpoint in preclinical studies, there is little to no consensus as to the most appropriate assessments. A variety of assays have been used in young-adult models using primarily acute time points and have been shown to correlate with infarct volume but have never been applied to an aged thromboembolic model with long-term assessments. We demonstrate herein the applicability of the mNSS but undoubtedly other tests may want to be explored, particularly those that are sensitive to higher order functions such as cognition and complex sensorimotor behaviors.

### Limitations

There is an inherent bias within preclinical ischemic stroke work, particularly in aged animals, with regards to long-term survival studies as animals with presumably the largest infarctions expire prior to assessment at delayed time points, thus preventing acquisition of data. Animals that survive for week’s post-MCAO generally have smaller infarcts, as was the case within this work. Specifically, animals surviving to 21 days had a total mNSS score of 6.3871 ± 2.6793 on day 1 post-MCAO, consistent with a mild to moderate impairment rather than a severe deficit. The lack of rodents exhibiting more severe deficits chronically post-stroke is likely a product of minimal post-operative care relative to the clinical scenario. Patients afflicted with stroke are often monitored continuously both during and after ischemia with extensive critical care, intervention, and rehabilitation support [30], a striking contrast to preclinical studies in which pain medication and nutritional support are applied but little else. Consequently, severely afflicted patients may survive whereas this is unlikely in animals.

Similarly, the eloquence of human behavior, a vital component of post- stroke assessment in which patients are asked to communicate, both verbally and written, as well as perform simple drawings and motor tasks, allows for the observation of abilities the emphasize primarily motor, sensory, or cognition. In rodents most, if not all, functional assessments require extensive motor ability. As such, when significant tissue damage occurs within the striatum or motor-associated parts of the cortex, assessments may be impaired due to reduced movement despite the fact that sensory and cognitive abilities are intact.

Without access to small animal imaging, this work required histological quantification of infarct volumes and therefore necessitated animal sacrifice. As such, we were unable to assess how acute infarct volume influences chronic functional assessments. Future studies may want to address this question in a clinically relevant model of ischemia.

## Figures and Tables

**Table 1:** Spearman correlation coefficients between histologic measures (edema index, cortical, striatal, and total infarct volume) and functional assessments (mNSS, grip strength, inclined, plane, and activity monitoring) at 24 hours post-MCAO. A P < 0.05 was considered statistically significant. Data is presented as spearman correlation coefficient (top) and P value (below).

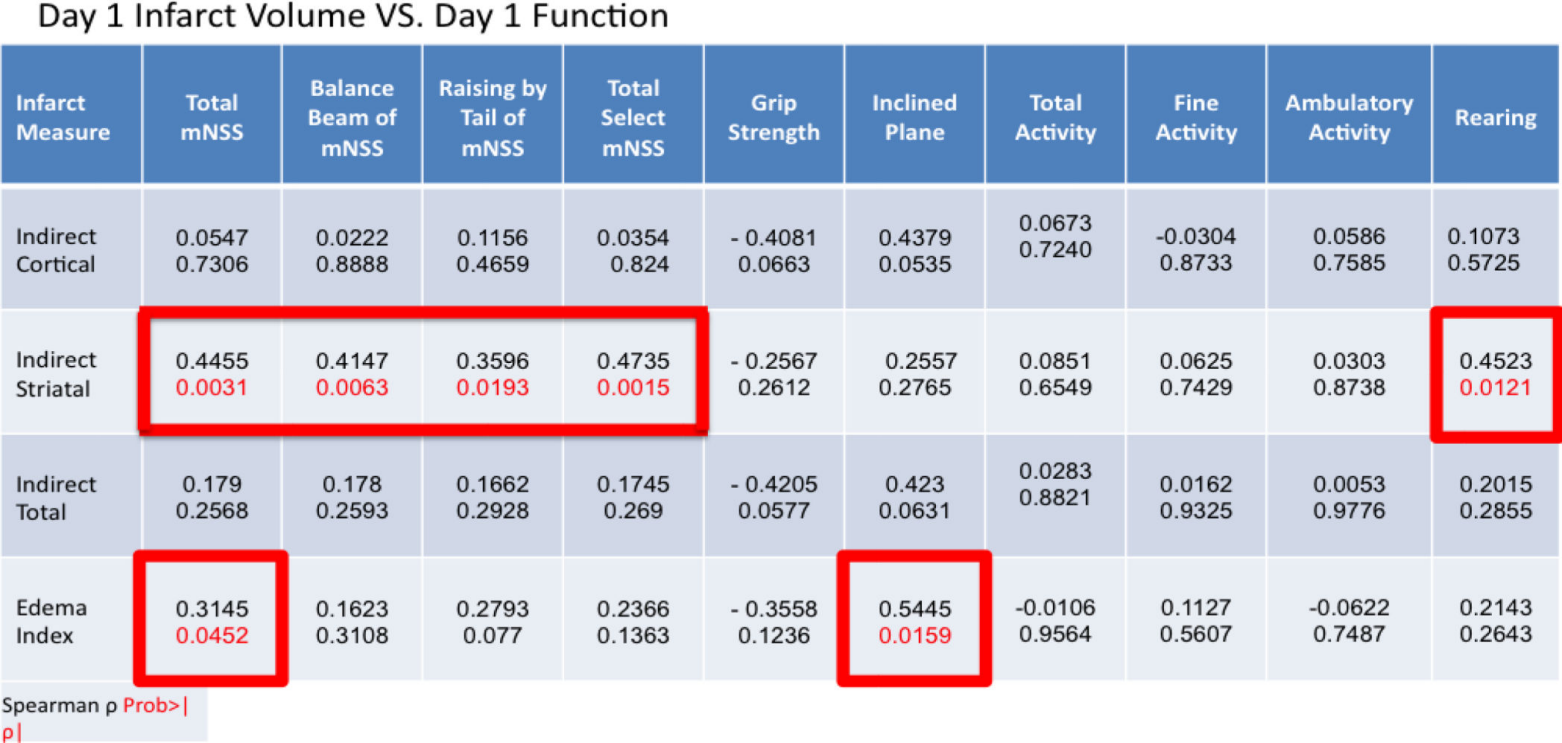

**Table 2:** Spearman correlation coefficients between histologic measures (edema index, cortical, striatal, and total infarct volume) and functional assessments (mNSS, grip strength, inclined plane, and activity monitoring) at 21 days post-MCAO. A P < 0.05 was considered statistically significant. Data is presented as spearman correlation coefficient (top) and P value (below).

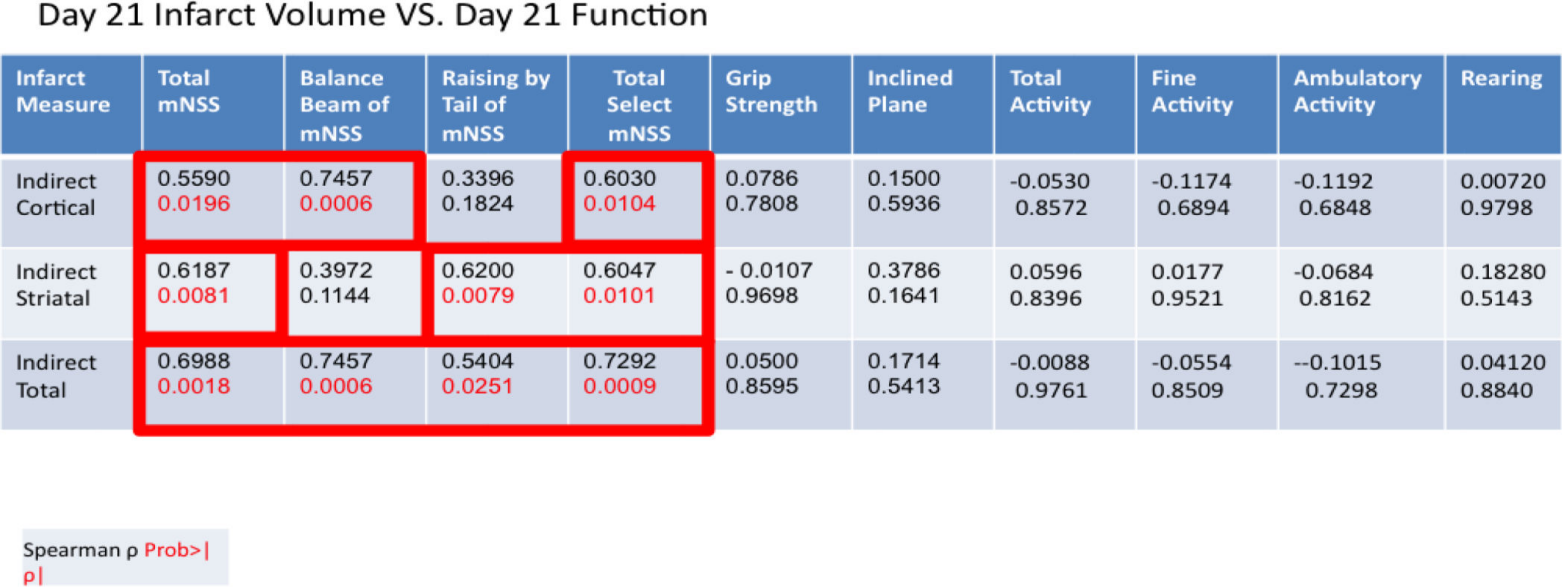

**Table 3:** Spearman correlation coefficients between histologic measures (edema index, cortical, striatal, and total infarct volume) determined at 21 days post-MCAO and functional assessments (mNSS, grip strength, inclined plane, and activity monitoring) conducted at 24 hours post-MCAO. A P < 0.05 was considered statistically significant. Data is presented as spearman correlation coefficient (top) and P value (below).